# RNA Metabolism Guided by RNA Modifications: The Role of SMUG1 in rRNA Quality Control

**DOI:** 10.3390/biom11010076

**Published:** 2021-01-08

**Authors:** Lisa Lirussi, Özlem Demir, Panpan You, Antonio Sarno, Rommie E. Amaro, Hilde Nilsen

**Affiliations:** 1 Department of Clinical Molecular Biology, University of Oslo, 0318 Oslo, Norway; lisa.lirussi@medisin.uio.no (L.L.); panpan.you@medisin.uio.no (P.Y.); 2 Department of Clinical Molecular Biology (EpiGen), Akershus University Hospital, 1478 Lørenskog, Norway; 3 Department of Chemistry and Biochemistry, University of California San Diego, La Jolla, CA 92093, USA; odemir@ucsd.edu (Ö.D.); ramaro@ucsd.edu (R.E.A.); 4 Department of Clinical and Molecular Medicine, Norwegian University of Science and Technology, NTNU, NO-7491 Trondheim, Norway; Antonio.sarno@sintef.no; 5 SINTEF Ocean AS, 7010 Trondheim, Norway

**Keywords:** SMUG1, rRNA processing, modified bases

## Abstract

RNA modifications are essential for proper RNA processing, quality control, and maturation steps. In the last decade, some eukaryotic DNA repair enzymes have been shown to have an ability to recognize and process modified RNA substrates and thereby contribute to RNA surveillance. Single-strand-selective monofunctional uracil-DNA glycosylase 1 (SMUG1) is a base excision repair enzyme that not only recognizes and removes uracil and oxidized pyrimidines from DNA but is also able to process modified RNA substrates. SMUG1 interacts with the pseudouridine synthase dyskerin (DKC1), an enzyme essential for the correct assembly of small nucleolar ribonucleoproteins (snRNPs) and ribosomal RNA (rRNA) processing. Here, we review rRNA modifications and RNA quality control mechanisms in general and discuss the specific function of SMUG1 in rRNA metabolism. Cells lacking SMUG1 have elevated levels of immature rRNA molecules and accumulation of 5-hydroxymethyluridine (5hmU) in mature rRNA. SMUG1 may be required for post-transcriptional regulation and quality control of rRNAs, partly by regulating rRNA and stability.

## 1. Introduction

A wide variety of functional base modifications are present in cellular RNA in addition to the regular four ribonucleosides. Over 160 known chemical modifications that modulate the structure and function of RNA molecules have been described [1,2,3,4,5,6,7]. Although most of the modifications described so far are found in abundant non-coding RNAs (ncRNAs), such as transfer (tRNAs) and ribosomal RNAs (rRNAs), recent advances in enrichment/capture techniques coupled with next-generation sequencing strategies have revealed an increasing number of different modifications both on coding and non-coding RNAs. Thus, all RNA classes, including messenger (mRNAs) and small nuclear RNAs, contain base modifications. For example, N6-methyladenosine (m6A), N1-methyladenosine (m1A) [8], 5-methylcytidine (m5C) [9,10,11], 5-hydroxylmethylcytidine (hm5C) [12], and inosine [13] are found in mRNA [14]. Base modifications introduced enzymatically at defined positions change RNA function at several levels. Here, we will first give an overview of the main rRNA modifications and RNA quality control mechanisms and then discuss recent developments implicating the SMUG1 DNA-glycosylase in rRNA biogenesis. In SMUG1 knock-down cells, immature and mature rRNAs accumulated 5-hydroxylmethyluridine (hm5U), a base modification recognized by SMUG1, pointing to SMUG1 as a possible new enzyme involved in the regulation of rRNA.

## 2. Ribosomal RNA Modifications and Their Biological Functions

**Figure 1:**
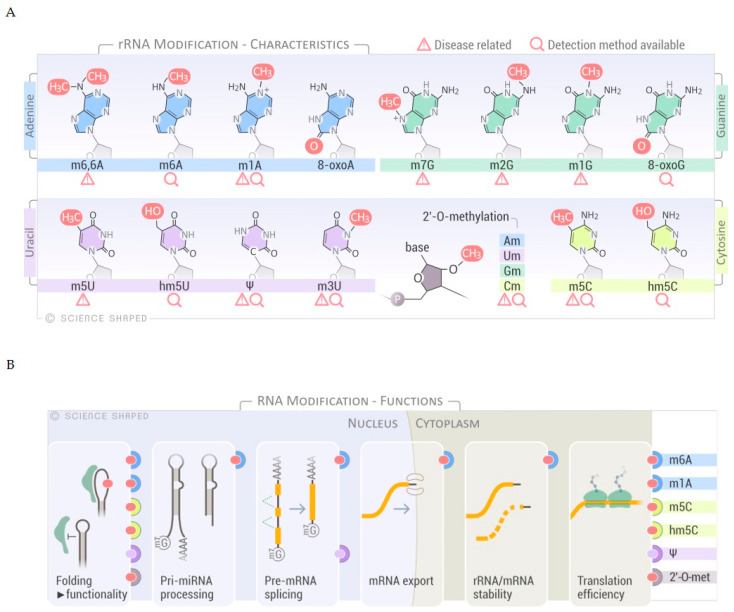
Overview of RNA modifications, substrates and functions. (**A**) Selected spectrum of RNA chemical modifications found in ribosomal RNA (rRNA). Disease relationships and sequencing methods are shown. (**B**) Schematic representation of the different functions of the selected RNA modifications. The modifications known to have a role in specific RNA processes are shown on the right-hand side of the panel.

In eukaryotic rRNA, 2% of all rRNA nucleotides are modified [6]. Several modifications have been mapped: These include enzymatically deposited base modification, exemplified by pseudouridines (Ψ), spontaneously introduced base modifications, e.g., oxidized bases, and residues methylated at the ribose phosphate backbone, such as 2′-*O*-methylation of ribose moieties (Am, Gm, Cm, and Um) (Figure 1A). Deposition of RNA modifications is regulated at several levels. In human cells, the majority of the small nucleolar RNA complex (snoRNP)-guided modifications, e.g., pseudouridylation and 2′-*O*-methylation, occur at early ribogenesis steps [6]. Only a few snoRNAs have been shown to target late pre-ribosomal intermediates where the activity of other factors, such as the RNA helicases, is required for structural rearrangements of the target to allow the snoRNAs to access the substrate [6,15]. On the other hand, other base modifications occur at later stages of ribosomal maturation, although the specific stage and timing has not been elucidated [6,16].

Further regulation of RNA modification is achieved through a specific association of the target RNA molecule and guide snoRNA in determined cellular locations, a molecular matchmaking that has been extensively described for pseudouridylation [17,18,19]. Modifications on rRNA are mainly concentrated in functional regions of the ribosome such as the peptidyl transferase center, the intersubunit interface, and the decoding and tRNA binding sites [6,20,21]. Modifications regulate not only the efficiency and accuracy of translation, as a consequence of ribosomal structure and function, but also rRNA processing and cleavage [6,22]. These modifications alter the structure/conformation and stability of the rRNAs due to changes in the molecular interactions within functional domains and distant regions of the ribosome. In addition, rRNA modifications may change the affinity of ribosomes to specific mRNA structures (i.e., internal ribosome entry sites), thereby governing the protein synthesis of a particular subset of mRNAs [6,23]. Interestingly, a role for rRNA modifications in ribosome heterogeneity by altering the ribosomal activity in response to environmental stressors has recently emerged, expanding the functions of modified bases in rRNA regulation to include fine-tuning of the translation cycle and modulating gene expression in response to external cues (Figure 1B) [6].

Several reports connect defects in the rRNA modification machinery, which encompasses snoRNAs and protein components of the snoRNP complexes or stand-alone rRNA-modifying enzymes, with genetic diseases and cancers. However, it is still unclear whether pathogenic effects are driven by the lack of modification per se [6].

Here, a selected set of known rRNA modifications and their functions are presented (for a comprehensive list of all the rRNA modifications and the sequencing methods used for their detection, the reader is redirected to [24]).

### 2.1. Pseudouridylation

Pseudouridine is considered the fifth ribonucleoside due to its abundance, and it is found in both the large and small subunits of the ribosome [6,19,25,26]. The role of Ψ in rRNA is still under debate, but it is classically described to improve base stacking due to increased backbone rigidity and to stabilize the secondary and tertiary structures for ribosomal subunit association [22,25,27]. 

Pseudouridine is enzymatically introduced by the isomerization of uridine. In humans, the main pseudouridylase is dyskerin (DKC1) [28]. DKC1 exerts its function as part of a ribonucleoprotein (RNP) complex, which consists of four core proteins (GAR1, NHP2, and NOP10 in addition to DKC1) and a short RNA molecule (H/ACA snoRNA). The H/ACA snoRNA contains a conserved 5′-ANANNA-3′ sequence called “hinge box” (or H-box) and a sequence of three nucleotides (ACA) present at its 3′-end (called ACA-box). This snoRNA functions as a guide RNA that defines the residue to be modified through base pairing in the “pseudouridylation pocket,” while the protein complex ensures the correct positioning of the target nucleotide [6,27,29]. Even though most of the pseudouridines in rRNA are modified by the H/ACA-snoRNA-guided machinery, a contribution from stand-alone human pseudouridine synthases (i.e., PUS1 and PUS7) cannot be completely excluded [6,25]. How specificity is achieved for PUS enzymes remain to be discovered [30]. 

Pseudouridine-related enzymes are implicated in human diseases. X-linked dyskeratosis congenita (X-DC), a severe disorder characterized by bone marrow failure, lung fibrosis, and increased susceptibility to cancer, is caused by mutations in *DKC1*. Patients present lower levels of Ψ compared to the healthy controls that may ultimately impair internal ribosome entry site (IRES)-mediated translation of a subset of mRNAs, such as TP53 and CDKN1B [6,18,20,23,31,32,33,34]. Illustrating the complex pathogenicity of pathways involving RNA modification enzymes, many symptoms of X-DC patients are related to the function of DKC1 in telomere maintenance and not to the deposition of pseudouridines in rRNA per se [22,35,36,37]. Two PUS enzymes are associated with human diseases; *PUS1* and *PUS3* mutations are found in patients with the mitochondrial disease MLASA (Mitochondrial myopathy, lactic acidosis and sideroblastic anemia) [38] and with intellectual disability [39], respectively. 

While early work was restricted to studies of pseudouridine in highly abundant long-lived RNA species due to limited sensitivity and specificity of the methods, recent technological developments in the targeted sequencing of RNA modifications have allowed the identification of pseudouridylated modifications at single-nucleotide resolution present in sub-stoichiometric amounts in non-coding RNA as rRNA, tRNA, and small nuclear RNA. The most common sequencing technique for the detection of pseudouridines is based on the derivatization of Ψ with carbodiimide and mutation insertion or block of reverse transcription during high-throughput sequencing [18,40,41,42]. Different efficiencies in carbodiimide incorporation makes this technique semi-quantitative. To alleviate this limitation, a novel method based on hydrazine/aniline cleavage was recently developed for systematic mapping and absolute quantification of Ψ, where the signals obtained by negative hits correspond directly to Ψ residues, protected from the hydrazine-dependent cleavage [43].

### 2.2. 2′-O-Methylation

Ribose 2′-*O*-methylation (2′-*O*-Me) at any nucleotide (Am, Gm, Um, and Cm), is another highly abundant modification with more than 100 sites reported for human rRNA (Figure 1A,B) [6,44]. 2′-*O*-Me might be involved in stabilizing the secondary and tertiary structures of rRNA, essential for ribosomal function (Figure 1B). As demonstrated on synthetic substrates, 2′-*O*-Me impaired the stability and flexibility of the stem-loops by preventing hydrolysis of the phosphate backbone and favoring an endo conformation at the 3′-end [22,45]. These methylations are formed by either stand-alone enzymes or by C/D-box snoRNP complexes that base-pair with the pre-rRNA and re-direct the RNA modifying enzyme to the specific target residue, the same principle as described for pseudouridylation guided by H/ACA box snoRNP [44]. The C/D box RNA has a bipartite structure containing a C-box (5′-RUGAUGA-3′, where R is a purine), a D-box (5′-CUGA-3′) at both ends, and related C′- and D′-boxes in the internal regions. The spacer regions between the boxes contain a guide sequence that can range from 10 to 21 nucleotides [46]. The proteins that form the RNP complex (NOP56, NOP58, and 15.5K) facilitate base-pairing and positioning of the catalytic site of the methyltransferase fibrillarin (FBL) to its target [6]. Although only 10 nucleotides form guide–substrate duplexes, the extensive base-pairing enhances the specificity of target recognition and prevents misfolding of the rRNA by sequestering the target [6,46]. Interestingly, a recent study in *Saccharomyces cerevisiae* indicates that a subset of snoRNAs can use a single guide to induce multiple modifications in the target region by forming two different snoRNP complexes that differ with respect to the positioning of the protein components (NOP56 and FBL). This mechanism may also be possible in other eukaryotes, which could increase the complexity of rRNA modifications without the requirement of additional snoRNAs [47]. 

The development of new high-throughput approaches has substituted laborious methods based on RNase H cleavage and retrotranscription. Through detection and systematic mapping of 2′-*O*-Me in different samples, it was shown that hypomodified regions lie peripherally on the 3-D structure of the ribosomes while the functional centers are heavily modified [48,49,50,51,52,53,54]. These methods confirmed the co-existence of distinct subsets of ribosomes that are only partially modified and may potentially exert specific functions [23,50,54]. Changes in 2′-*O*-Me profiles in rRNA have been linked to ribosomopathies such as Treacher Collins syndrome and cancer susceptibility [6,55,56]. 

### 2.3. Other Base Modifications

The emergence of new technology for mapping RNA modifications has led to the unequivocal identification of less abundant modifications and brought new understanding of their functions and their link with human diseases, although many aspects of their biology are still unclear [24,57]. 

In humans, N6-methyladenosine (m6A) has been found only in three sites of rRNA, at position A1832 in 18S and at position A4190 and A4220 in 28S rRNA [1,58,59,60,61]. The methyltransferase ZCCHC4 was identified as the enzyme responsible for the deposition of m6A at position A4220 in 28S rRNA. ZCCHC4 mainly methylates 28S rRNA and decreased levels of this modification negatively affect global translation, reducing cell proliferation. Interestingly, ZCCHC4 is overexpressed in hepatocellular carcinoma, suggesting a link between rRNA m6A modifications and tumor biology [60]. Recently, a heterodimer formed by METTL5 and TRMT112 was identified as the enzymatic complex responsible for the deposition of m6A at A1832 on 18S rRNA, through extrusion of the adenosine residue from the DNA helix [62,63]. Functions of m6A in other positions of rRNA, and the enzymes responsible for their deposition, are still unknown. The identification of m6A residues is challenging due to nearly identical chemical properties of the modified and unmodified nucleotides and the preferential methylation only within DRA*CH sequence contexts (D = A, G, or U; R = purine; A* = methylatable A; H = A, C, or U) [64,65]. Mapping techniques, such as m6A-Seq and miCLIP have been developed to overcome these challenges [66,67,68].

Further methylation of m6A to N6,N6-dimethyladenosine (m6,6A) has been identified in two sites of 18S rRNA (A1850 and A1851) as result of the combined action of the human methyltransferase DIMT1L and hDIM2 [4,69,70]. 

N7-methylguanosine (m7G) modification at G1639 in 18S rRNA by the WBSCR22–TRMT112 complex occurs prior to dimethylation of A1850/A1851, and the two modifications seem to exert similar functions in pre-rRNA processing. In fact, the binding of both DIMT1L and WBSCR22–TRMT112 to rRNA, but not their catalytic activity, is required for pre-rRNA processing. Their depletion affects the kinetics of 18S rRNA synthesis, as a result of defects in cleavage at sites A0 and 1 and site 2, respectively [69]. These modifications lie in conserved sites of the ribosome (decoding site and a ridge between the P- and E-site tRNAs). In bacteria, they are essential for a packing interaction near the A-site, affecting rRNA structure and ultimately translation efficiency. In humans, their functions have not been determined, but it has been proposed that m6,6A and m7G may function in rRNA quality control, maturation surveillance, and nuclear export of the pre-ribosomes [69,70].

N1-methyladenosine (m1A)-modified RNA was first shown in yeast, and the human counterpart was recently discovered. m1A is found in the large subunit of the ribosome at position A1322 in 28S rRNA, and it is introduced by nucleomethylin (NML). It affects the local structure of the ribosome, promoting proper conformation of the 60S and ultimately translation [71,72]. Two different methods for the detection of m1A have been described and they rely on the ability of m1A to stall transcription [71,73,74,75].

8-Oxo-7,8-dihydroguanosine (8-oxoG) was recently found in rRNA, arising from spontaneous oxidation, but no data are available on the oxidation of specific rRNA residues [76,77]. Sequencing methods to identify oxidized ribonucleotides are under development [78]. An in vitro mutagenesis study in *Escherichia coli* showed that the effect of oxidation (both 8-oxoG and 8-oxo-7,8-dihydroadenosine, 8-oxoA) on protein translation is mediated by specific residues close to the peptidyl transferase center that seem to be hotspots for oxidation [77]. Ribosome oxidation is associated with ribosomal dysfunction, altered protein translation, and loss of neurons. Increased levels of 8-oxoG-containing rRNAs are correlated with neurodegenerative conditions, such as Alzheimer’s disease [77], but this likely reflects RNA oxidation as a consequence of the oxidative stress that often accompanies this condition. Guanosine is the most common oxidative lesion having the lowest redox potential of all the four bases [79]. Thus, whether 8-oxoG has a functional role as an RNA modification or reflects RNA damage has not been clarified.

5-Methylcytidine (m5C) was mapped to two sites (C3782 and C4447) in 28S rRNA in humans, and no evidence is available so far for m5C modification in 18S rRNA. The enzymes responsible for these modifications are NSUN5 and NSUN1 for C3782 and C4447, respectively. m5C is important for the stability of rRNA structures by promoting base stacking and thermal stability of hydrogen bonding [80]. In yeast, loss of m5C at C3782 induces structural changes and ribosome instability that affects protein translation under stress. The role of m5C at C4447 in ribosomal function remains unknown [9,61,64,81,82,83,84,85]. Many experimental approaches have recently been developed to detect m5C sites [9,10,11,86]. 

The oxidized derivative of m5C, 5-hydroxymethylcytidine (hm5C), has been identified in RNA. It was proposed that m5C might represent a transient intermediate or that its oxidized products may be in a dynamic equilibrium with m5C in RNA [12,24,85,87].

5-Methyluridine (m5U), 5-hydroxymethyluridine (hm5U), N1-methylguanosine (m1G), N2-methylguanosine (m2G), and N3-methyluridine (m3U) have been described in bacterial rRNA and identified in both human 40S and 60S subunits, but further studies are needed to conclude whether these modifications may have regulatory roles in rRNA metabolism [24,57,88].

The development of techniques for the detection of specific modifications at single-nucleotide resolution reinvigorated studies of these post-transcriptional modifications and their role in RNA structure/function [68,89,90,91,92,93,94]. However, the downstream bioinformatic analyses may not always be reproducible in all the laboratories, so work is being done to improve the analysis of the RNA “epistructurome” of the RNA Framework, an all-in-one toolkit for the analysis of most Next-Generation Sequencing (NGS)-based RNA structure probing and post-transcriptional modification mapping experiments (http://www.rnaframework.com) [95].

## 3. rRNA Processing and Maturation

**Figure 2:**
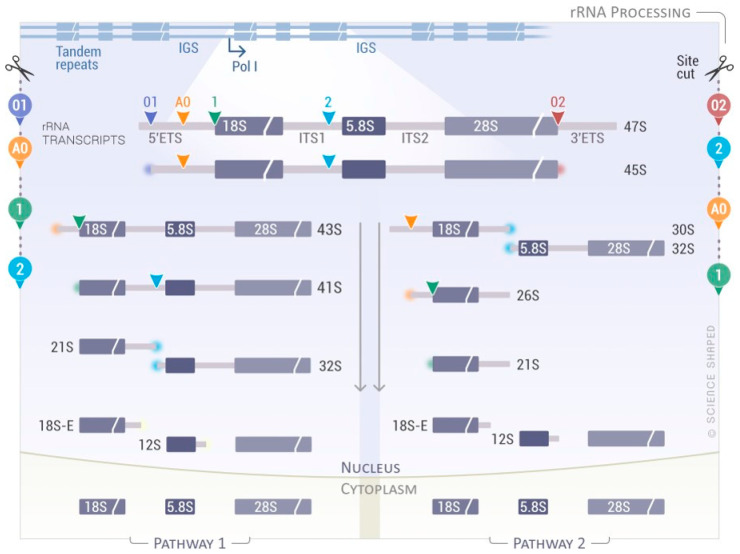
Simplified rRNA processing pathway in eukaryotes. RNA Polymerase I (Pol I) transcribes a long primary transcript (47S pre-rRNA) from loci containing hundreds of rDNA copies. This transcript contains the sequences for the mature 18S, 5.8S, and 28S rRNAs flanked by external (5′- and 3′-ETS) and internal spacers (ITS1 and ITS2), which are enzymatically removed by endo- and exonucleases. Depending on the cleavage sites used and the kinetics, two main pathways can be depicted forming different precursors. Cleavage of the 45S rRNA precursor starts either in the 5′-ETS at cleavage site 1 (Pathway 1, left side) or in the ITS1 sequence at cleavage site 2 (Pathway 2, right side). The cleavage sites used for each pathway are shown on the side.

Ribosome biogenesis and assembly of the small and large subunits of the eukaryotic ribosome takes place in the nucleus before its final maturation in the cytoplasm. It requires the association of 80 ribosomal proteins (RPs) with four distinct ribosomal RNAs. The small subunit (40S, SSU) is formed by the association of the 18S rRNA with 33 RPs; the large subunit (60S, LSU) contains the 5S, 5.8S, and 25S/28S rRNAs associated with 47 RPs [96]. The ribosomal genes are arranged as direct head-to-tail tandem ribosomal DNA (rDNA) repeats at the nucleolar organizer regions (NORs), and they are present in several copies within eukaryotic genomes (>200 rRNA genes/genome of five distinct chromosomes). Only a fraction of these genes is actively transcribed [97]. rRNA biogenesis starts in the nucleolus where RNA polymerase I (RNAPI or PolI) transcribes a long primary transcript that has to be processed in order to produce the mature rRNAs (18S, 5.8S, and 25S/28S rRNAs) (Figure 2). The primary transcript (47S) comprises the mature rRNAs separated by the internal transcribed spacer 1 (ITS1) and 2 (ITS2) flanked by the 5′- and 3′-external transcribed spacers (5′- and 3′-ETS) (Figure 2). The primary transcript is sequentially cleaved via a complex sequence of endonucleolytic and exonucleolytic cleavages (Figure 2) [96,98,99,100]. The 47S rRNA is cleaved at the 5′- and 3′-ends (sites 01 and 02, respectively) to form the 45S precursor that can be processed via two main pathways (pathway 1 and 2), depending on the cleavage sites used. In pathway 1, processing starts at the 5′-end of the molecule with the cleavage at sites A0 and 1, forming 41S rRNA followed by successive trimming at site 2 into 21S and 32S pre-rRNAs. In pathway 2, the cleavage begins at site 2, located within the ITS1. The newly generated 30S pre-rRNA is further processed directly to 21S pre-rRNA through cutting at the A0 and 1 sites, or via an intermediate form called 26S pre-rRNA, where the cleavage at these sites is uncoupled (Figure 2) [96,98,99,100]. Intriguingly, a role for the exosome and DIS3L2/ERI1 in the 5.8S maturation steps via the formation of a cytoplasmic precursor, named 7SB, was recently demonstrated [101]. 

For an exhaustive description of the rRNA processing pathways, the reader is redirected to recent review articles [96,98,99,100,101].

## 4. RNA Quality Control Mechanisms

**Figure 3:**
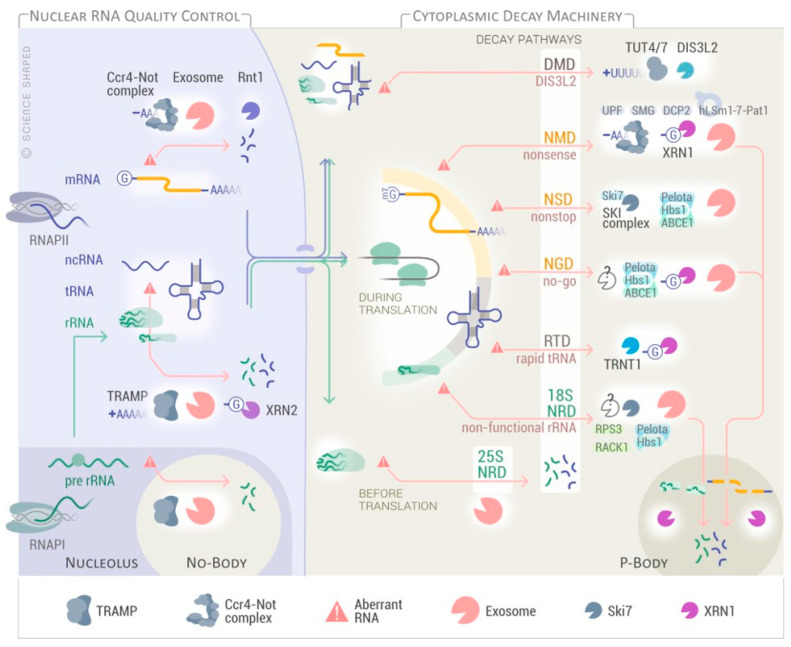
RNA degradation pathways in eukaryotes. RNA quality control mechanisms and their cellular compartments for aberrant mRNA, tRNA, rRNA, and ncRNA in eukaryotic cells are depicted. NMD, nonsense-mediated mRNA decay; NGD, no-go decay; NSD, non-stop decay; NRD, non-functional rRNA decay; RTD, rapid tRNA decay; DMD; DIS3L2-mediated decay.

Eukaryotes possess multiple quality control mechanisms that operate in different cellular compartments to eliminate specific classes of RNA molecules (Figure 3). There are two major strategies of RNA degradation: specialized RNA nucleases (endonucleases or 5′-3′ exonucleases) and the exosome, which is an RNA degradation factory that, in many ways, resembles the proteasome [102]. The exosome is a multiprotein complex, which is equipped with three different ribonuclease activities: endonuclease and 3′-5′ exonuclease activities are supported by the Dis3 subunit and a second 3′-5′ exonuclease activity by the Rrp6 subunit [103]. The exosome is responsible for processing, degradation, and regulated turnover of all classes of RNA in eukaryotes [104,105]. These modes may be engaged non-exclusively and operate together. Cytoplasmic mRNAs subjected to degradation are either deadenylated prior to 3′ to 5′ degradation by the exosome [106], or decapped and then degraded by 5′-3′ exonuclease 1 (XRN1) [107]. Deadenylation involves the collaboration between one of the two deadenylases CCR4 and CAF1 of the Ccr4–Not complex and the related deadenylase Pan2/3 [108]. The removal of the poly(A) tail is followed by degradation by the exosome complex.

### 4.1. mRNA Surveillance Pathways

**Figure 4:**
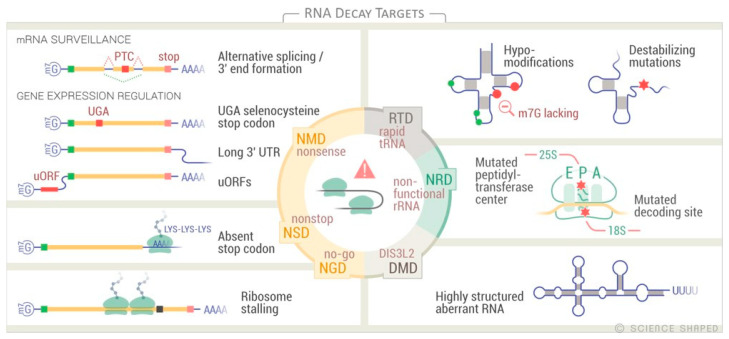
RNA decay target features in eukaryotes. Summary of features for the decay targets. NMD, nonsense-mediated mRNA decay; NGD, no-go decay; NSD, non-stop decay; NRD, non-functional rRNA decay; RTD, rapid tRNA decay; DMD; DIS3L2-mediated decay.

Eukaryotic cells present three main cytoplasmic RNA quality control processes that are activated in response to defects in translation: the nonsense-mediated decay (NMD), the no-go decay (NGD), and the non-stop decay (NSD) pathways (Figure 3 and Figure 4). All of these pathways use the ribosome as the initial recognition machinery for defective mRNAs [109,110]. 

NMD promotes the degradation of mRNAs undergoing premature translation termination due to the generation of premature termination codons (PTCs) (Figure 4). NMD functions in mRNA quality control by preventing the synthesis of possibly harmful truncated proteins. It also plays an important role in regulating gene expression via the degradation of natural mRNAs that present features specifically recognized by the NMD machinery such as translated upstream open reading frame (uORF), atypically long 3′-untranslated regions (3′-UTR), and UGA selenocysteine stop codons (Figure 4) [111]. PTCs can arise in mRNAs through different mechanisms such as transcription errors, mutations, and alternative splicing events that can expose intronic stop codons or cause frame shifts within the coding region [112]. When the ribosome stalls at a PTC, Upf (Upf1, Upf2, and Upf3), and Smg proteins associate with the defective mRNA, targeting it to RNA degradation via an endonucleolytic cleavage, decapping, or deadenylation (Figure 3) [112,113]. NMD also actively represses the recruitment of newly formed ribosomes to the defective mRNA [114,115].

NGD targets transcripts that stall the ribosome, i.e., secondary structure, rare codons, and depurination sites (Figure 4) [116,117,118]. In NGD, the Pelota–Hbs1 complex binds the A site of the stalled ribosome in a codon-independent manner and starts an endonucleolytic cleavage by unknown nucleases. This endonucleolytic cleavage is followed by the degradation of the defective mRNAs via the exosome (Figure 3) [118,119,120,121,122]. Since NGD targets also sequester functional ribosomes from the translating pool, several factors, such as eRF3 and eRF1 paralogs (Pelota and Hbs1) and ABCE1 (Rli1 in yeast), have additional roles in dissociating ribosome subunits and peptidyl-tRNAs, thereby accelerating the recycling of the stalled ribosomes (Figure 3) [123,124,125]. 

NSD detects mRNAs lacking a stop codon due to mutations or ribosomes bypassing the normal stop codon (Figure 4). mRNAs are targeted to the exosome for degradation in response to ribosome stalling on the poly-Lys encoding poly-A tails [126]. Additional substrates for NSD include prematurely aborted or polyadenylated transcripts and mutated transcripts that affect the stop codon (Figure 4) [127]. In addition to the exosome activity, the NSD requires Hbs1–Pelota, the Ski7 protein, and the Ski complex (comprising the DEVH-box RNA helicase Ski2, Ski3, and Ski8) that physically and functionally interact with the exosome. Degradation via NSD does not require deadenylation [109,123,127]. 

Nuclear mRNAs are also subjected to degradation when processing or export are altered. In these cases, mRNAs are degraded by the nuclear exosome or cleaved by the endonuclease RNT1 and then degraded by the nucleases Rrp6 and Rat1 in yeast (XRN2 in human) [128]. The Ccr4–Not complex may be also required to tether misprocessed mRNAs to sites of transcription to prevent their export or act as a scaffold to recruit the exosome to destroy them [129].

### 4.2. rRNA Quality Control

rRNAs may be degraded both in the cytoplasm and in the nucleus. Upon translation failure, cytoplasmic rRNAs are degraded by a process referred to as non-functional rRNA decay (NRD) (Figure 3) [130]. Introduction of deleterious mutations, in either the 25S peptidyl transferase center or the 18S decoding site, leads to reduced stability and, consequently, downregulation of the modified rRNAs [130,131]. Interestingly, although both mutations in the 25S and in the 18S rRNAs result in defective or chemically damaged ribosomes, cells degrade them through two distinct and specialized pathways, the 25S NRD and the 18S NRD (Figure 3) [109,132]. 

The 25S NRD substrates, which accumulate around the nuclear envelope in perinuclear foci, are eliminated after export to the cytoplasm in a process involving the exosome [131]. The proteasome and the E3 ligase complex subunits Mms1 and Rtt101 are required for the initiation of rRNA degradation in the 25S NRD [133]. NRD-mediated degradation of defective 18S rRNAs that are distributed throughout the cytoplasm, depends on translation elongation and utilizes the same proteins as those participating in the NGD and NSD mRNA surveillance pathways, with an additional requirement of the recently described factors, Asc1 and Rps3 [134]. In both cases, the stalled translation complexes are processed by the exosome and then further degraded by XRN1 in P-bodies (Figure 3) [131]. 

In the nucleus, when rRNAs have defects during the maturation step, they can be polyadenylated by the Trf4–Air2–Mtr4 polyadenylation (TRAMP) complex before degradation by the nuclear exosome. The TRAMP complex adds short poly(A) tails to aberrant transcripts, forming a favorable substrate for the exosome and, thereby facilitates degradation [135]. In addition, non-coding small nuclear RNAs (snRNAs) and snoRNAs, whose turnover and/or processing needs the nuclear degradation machinery, are also affected by the Ccr4–Not complex, suggesting that Ccr4–Not connects TRAMP with the nuclear exosome for processing and/or degradation of their target RNAs [136,137]. Interestingly, investigation of the quality control mechanisms that detect and degrade irregular pre-rRNAs showed that pre-ribosome components, polyadenylated RNAs, TRAMP, as well as the exosome, concentrate in the subnucleolar structure termed No-body, in which pre-ribosome surveillance is likely to take place [138]. Other nuclear pre-rRNA surveillance quality pathways were initially described in *S. cerevisiae*, where in the absence of pre-rRNA dimethylation, for example, Dim1p blocks pre-rRNA processing steps required for the maturation of 18S rRNA [139]. The discovery of the functional human homologs DIMT1L and WBSCR22–TRMT112 and their role in rRNA-processing suggests that this pathway is conserved from yeast to humans [69].

### 4.3. tRNA Quality Control

tRNAs are long-lived RNA molecules. Defective tRNAs are degraded both in the nucleus and the cytoplasm via the combined action of the TRAMP complex, the nuclear exosome, and the rapid tRNA decay (RTD) pathway (Figure 3). These pathways ensure that the tRNAs are correctly structured, modified, and processed before translation [140]. They recognize and degrade tRNAs with aberrant structures and conformational changes that affect the tertiary fold (i.e., acceptor and T-stem regions) as well as hypo-modified tRNAs (Figure 4). Although the RTD pathway targets substrates mainly due to 5′-end exposure, it was recently found that this pathway also degrades tRNA variants with defects in the anticodon stem-loop, causing the accumulation of unspliced pre-tRNAs. These substrates are degraded via a distinct XRN1- and XRN2- independent RTD pathway [141,142,143,144,145]. The degradation via the RTD pathway is also facilitated by the addition of a second CCA triplet to the 3′-end of tRNAs by a CCA nucleotidyl transferase (Cca1 in yeast, TRNT1 in humans) [146,147]. The nuclear exosome and the 5′-3′ exoribonuclease XRN2, the latter as part of the nuclear RTD, can also remove precursor tRNAs that are processed too slowly and tRNAi Met lacking the m1A modification [140,142,148,149].

### 4.4. ncRNA Decay Pathway

Recent advances in high-throughput sequencing techniques have also improved our understanding of degradation of non-coding RNA. ncRNAs play key roles in cells, mostly through the formation of ribonucleoprotein complexes. The 3′-5′ exoribonuclease DIS3L2 is involved in the quality control of ncRNAs in the DIS3L2-mediated decay (DMD) pathway (Figure 3). DMD substrates are highly structured RNAs originating from incorrect processing from all three nuclear RNA polymerases such as rRNA, snRNA, snoRNA, tRNA, mRNAs (i.e., ARE-containing mRNAs), lncRNA, and transcripts from pseudogenes (Figure 4). Interestingly, DMD is the main degradation pathway for the sense and antisense transcription start site–associated sequence (TSSas). DMD targets are modified post-transcriptionally by two terminal uridyl transferases (TUTases), TUT4 (Zcchc11), and TUT7 (Zcchc6), which add a tail of uridines at the 3′-end of the ncRNAs, close to secondary structures, suggesting a conformational requirement for the recognition by TUTase. These terminal stretches are then recognized by DIS3L2, which rapidly degrades them. DIS3L2 is associated with polysomes, suggesting that some DMD substrates are targeted co-translationally [150,151,152,153,154,155,156,157,158].

## 5. Processing of Damaged RNA

While the processing of rRNA is well described, it is less clear which molecular event initiates the activation of the rRNA quality control machinery. In chemotherapy settings, it is well known that ribogenesis defects might be caused by the presence of modified or damaged bases in RNA. For example, in cells treated with 5-fluorouracil (5-FU), a synthetic analogue of uracil with a fluorine atom at the C5 position, 5-FU accumulates in RNA and RNA-mediated toxicity appears to be an important contributor to cytotoxicity [159]. Interestingly, several factors involved in 25S NRD, as the E3-Ubiquitin ligase components Mms1 and Rtt101, are involved in DNA repair, suggesting an overlap between the RNA and DNA surveillance mechanisms [109,133]. This link between DNA and RNA quality control systems has been reinforced by several reports indicating a role for the base excision repair (BER) proteins, the uracil glycosylase SMUG1, and the apurinic/apyrimidinic endonuclease APE1, in rRNA quality control under physiological conditions [76,88,160].

### 5.1. SMUG1 Structure and Function

DNA repair plays critical roles in the maintenance of genome integrity; around 150 proteins have so far been implicated in this process [161]. BER is a multi-step pathway that corrects a large number of spontaneous and environmentally induced lesions formed by oxidation, deamination, and alkylation of DNA. BER is initiated by DNA glycosylases that cleave the *N*-glycosylic bond between the damaged base and the 2′-deoxyribose of the nucleotide. The damaged base is then removed to generate an apurinic/apyrimidinic (AP) site that is recognized and cleaved by an AP endonuclease, APE1 or AP lyase [162]. After the initiation of BER, further processing may take place by short-patch or long-patch sub-pathways where a single nucleotide or a 2–10 nucleotide gap is generated and filled, respectively [163,164].

One of the most frequent lesions found in DNA is uracil (U), and it arises from the deamination of cytosine and misincorporation of dUMP instead of dTMP during replication. It can lead to G:C to A:T transition mutations [165,166]. The archetypal uracil-DNA glycosylases (UDGs), exemplified by *E. coli* and human uracil DNA *N*-glycosylases (UNG), can recognize and excise uracil in single- and double-stranded DNA regardless of the opposite base. In contrast, *E. coli* mismatch uracil-DNA glycosylase (MUG) or human thymine DNA glycosylase (TDG) only removes uracil in the U:G context [167]. As a monofunctional DNA glycosylase in the UDG family, SMUG1 excises uracil, oxidized uracil derivatives (e.g., 5- hydroxymethyluridine and 5-hydroxyuridine (ho5U)), and other oxidized pyrimidine (e.g., 5-formyluridine (f5U), 5-carboxyuridine (ca5U)), in both single- and double-stranded DNA [168,169].

**Figure 5:**
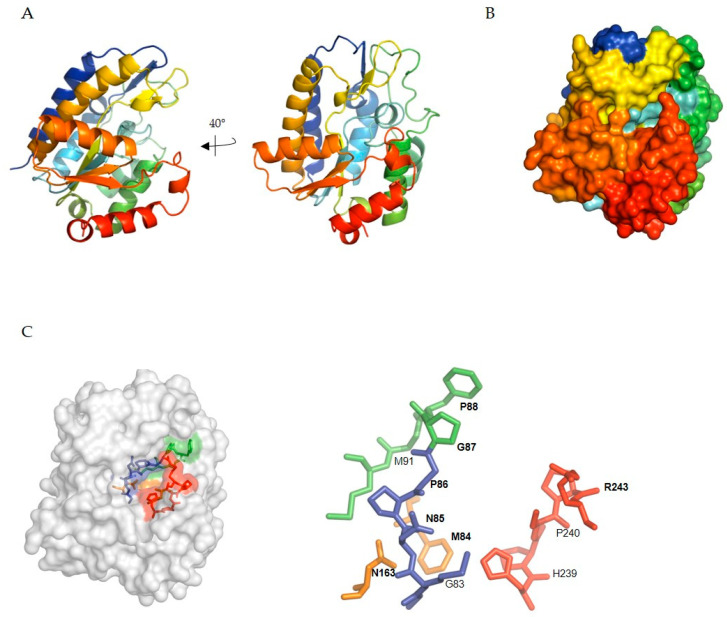
In silico modeling of human single-strand-selective monofunctional uracil-DNA glycosylase 1 (SMUG1) ∆25-270. (**A**) Secondary structure cartoon of hSMUG1 ∆25-270, rainbow colored blue → red from N- to C terminus. (**B**) Overview of active sites pocket and cleft shown as surface; rainbow colored blue → red from N- to C terminus. (**C**) Active site residues of hSMUG1 (left: overview; right: detail); G83-G87 (blue); P89-M91 (green); F98 and N163 (orange); H239-R243 (red). A homology model for hSMUG1 was built based on *Xenopus laevis* SMUG1 (PDB ID: 1OE4).

hSMUG1 is a single-domain protein with a typical α/β/α sandwich architecture (four-stranded parallel β-sheets bordered by α-helices) (Figure 5A,B). hSMUG1 has a substrate binding pocket formed by the C-terminal ends and the β strands (Figure 5A,B) [167]. hSMUG1 appears to interact rather non-specifically with DNA [170]. After initial binding, DNA glycosylases largely utilize base flipping to insert a damaged base into their active site binding pocket, where it is positioned for cleavage of the *N*-glycosylic bond. The conserved N-terminal GMNPG motif together with the C-terminal HPSPR motif form the pocket that positions the substrates for cleavage (Figure 5C, left) [171]. The region between Gly87 and Met91 recognizes the modified base in the C5 position via water bridges (uracil) or direct hydrogen bonding (ho5U, hm5U, f5U). In general, the specificity of DNA glycosylases is dictated through space restriction in the active site and in hSMUG1, Asn163, and Phe98, which discriminate against pyrimidine bases. DNA damage recognition and base flipping is performed by amino acids in the intercalation loop of hSMUG1 (amino acids 251–260) that open up the DNA double helix to facilitate base flipping whereas the extra-helical state is stabilized by inserting the Arg243 side-chain into the void of the flipped-out base. Two residues, Asn85 and His239, catalyze cleavage of the *N*-glycosylic bond (Figure 5C, right) [171,172]. 

No experimental structural data is available to verify whether a similar mechanism is utilized on RNA substrates, but there are reasons to suspect that RNA is processed in a similar manner: the C-terminal nucleic acid binding domain and the catalytic residue His239 are required for full activity on RNA substrates a well as DNA [88,173]. Our current data suggest that selection of RNA substrates for hSMUG1 is in part determined by the interaction with the DKC1 containing H/ACA RNP RNA substrates [88,173]. Interaction between SMUG1 and DKC1 involves two regions not required for catalysis, i.e., amino acids 25–35 and 220–233 [88]. Homology modeling suggested that the interaction surface did not comprise the nucleic acid binding domains of either protein [88]. hSMUG1 was able to process the deoxyribonucleoside hm5U in RNA but not ribo-uridine-containing substrates [88]. This suggests that there are likely additional interactions with the backbone that shape substrate specificity on RNA substrates that remain to be discovered. However, the available in vivo data strongly suggest that a modified base is a prerequisite for hSMUG1 action [88,173]. Hence, we suspect that hSMUG1 may be involved in a highly specialized rRNA quality control pathway [76,88]. However, much remains to be defined regarding the molecular mechanisms of how RNA targets are selected and processed. 

### 5.2. SMUG1 in Regulating a Highly Structured RNA

**Figure 6:**
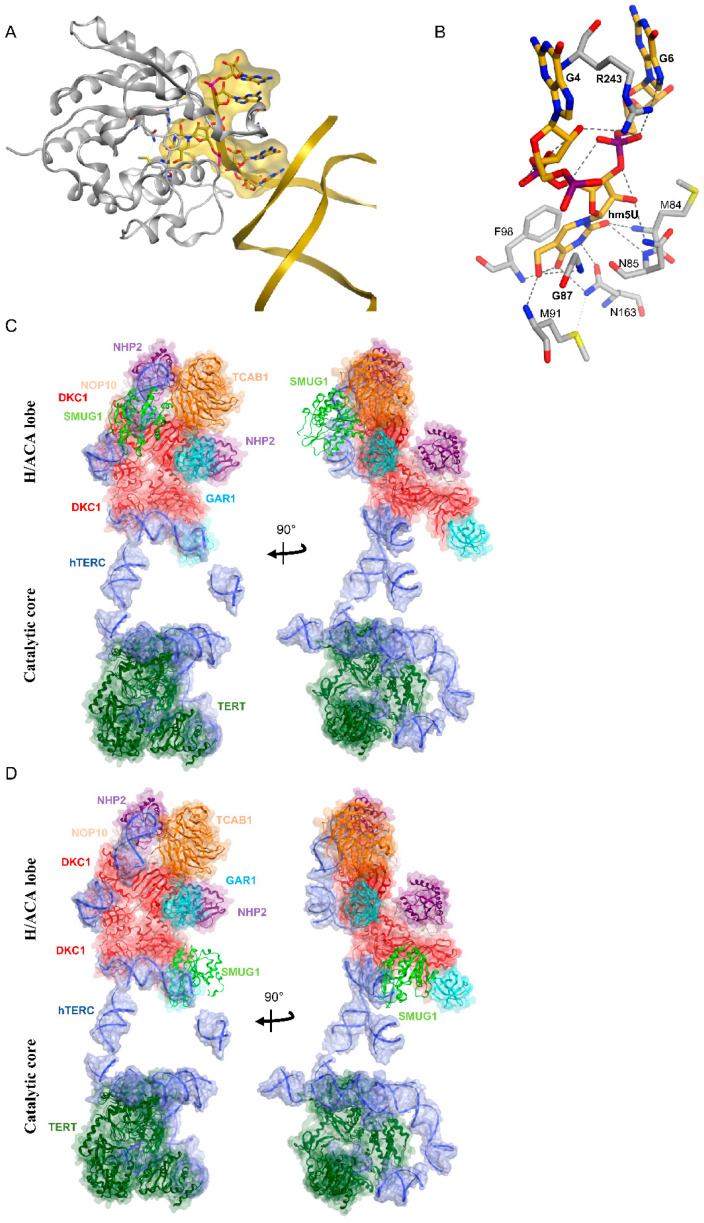
Computationally predicted binding pose of hSMUG1 to *TERC* and the human telomerase complex. (**A**) Homology model of hSMUG1 (shown in silver ribbons) bound to the fish *TERC* structure (shown in gold ribbons), which is computationally modified to mimic *hTERC* at the binding interface. The residues of hSMUG1 that interact with the flipped hm5U are shown in sticks with C atoms in silver. The flipped hm5U as well as the two nucleotides neighboring it on each side in *TERC* are depicted in sticks with C atoms in gold. Both for the residues and the nucleotides, N atoms are in blue, O atoms are in red, S atoms are in yellow, and P atoms are in magenta. The terminal 5 nucleotides of *TERC* are additionally highlighted depicting their molecular surface in gold. (**B**) Close-up view of panel A depicting the interaction of hSMUG1 active site with the flipped hm5U at position 220 and its neighboring nucleotides. Everything except the interacting residues is hidden to provide a clear view. The color code is the same as in panel A, except here P atoms are depicted in purple. Interactions are displayed as dotted lines. (**C**,**D**) Superimposing SMUG1 on the pdb structure of human telomerase complex cryo-EM. Our prior experimentally validated SMUG1–DKC1 complex structure is superimposed onto each of the two dyskerin (DKC1) units found in human telomerase. In both cases, a significant steric clash of SMUG1 with the rest of the human telomerase is predicted.

Recently, our group demonstrated a new role for hSMUG1 in controlling telomere maintenance through processing of the human telomeric RNA component (*hTERC*). We proposed that SMUG1 might regulate the presence of two modified bases (C323 and C445) positioned in the CR4/CR5 and H-box domains of *hTERC* [173]. *hTERC* is a highly structured non-coding RNA [174]. In an attempt to better characterize the interaction at the molecular level between hSMUG1 and *TERC*, we computationally predicted the binding pose of the homology model of hSMUG1 to the available structure of medaka *TERC* (PDB ID: 4026 chain E). As shown in Figure 6A,B, the flipped-out hm5U residue at C220 of fish *TERC*, corresponding to C323 in the *hTERC*, lies at the active site of hSMUG1. The residues involved in the binding with hm5U of fish *TERC* are highlighted in Figure 6B.

*hTERC* is one of the main components of the telomerase holoenzyme, together with the telomerase reverse transcriptase (hTERT) and the dyskerin complex (DKC1, NHP2, NOP1, and GAR1) [174,175]. Experimental data indicated that SMUG1 was required for the productive binding of DKC1 to *hTERC.* As the interaction between hSMUG1 and *hTERC* might differ when *hTERC* is present in the telomerase holoenzyme and when hSMUG1 interacts with DKC1, we decided to superimpose the SMUG1–DKC1 complex, previously characterized in [88], on the cryo-EM structure of the human telomerase complex [176]. Our SMUG1–DKC1 structure was superimposed onto each of the two DKC1 molecules present in the human telomerase (Figure 6C,D). In both the configurations, our modeling predicts a significant steric clash of hSMUG1 with the other components of the human telomerase, suggesting that SMUG1 most likely is not a constitutive component of the complex but dynamically interacts with the enzyme upon recognition of modified bases of *hTERC* (Figure 6C,D).

## 6. Discussion

**Figure 7:**
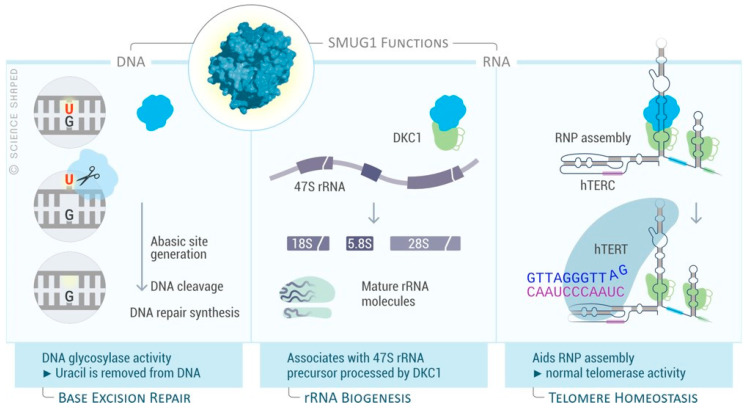
SMUG1 functions on DNA and RNA. SMUG1 role in DNA repair as DNA glycosylase in the base excision repair (BER) pathway and in RNA processing with functional relevance for ribosomal RNA biogenesis and telomere maintenance. Dyskerin 1, DKC1; human telomerase RNA component, *hTERC*; human telomerase reverse transcriptase, hTERT [178].

What happens to RNA molecules that are aberrantly modified? How are modified RNA molecules distinguished from damaged RNA molecules? Little is known about how cellular pathways manage to discriminate between these, in principle, different RNA modifications. Considerable progress has been made in the past years in our understanding of the biology of RNA modifications due to improvement in detection technology. The ability to detect different modifications of the same RNA molecule at the same time gives us a more comprehensive picture of the combinatorial role of these modifications in ribosome functions. RNA modifications not only act as structural modifications, but they also directly affect RNA functions, e.g., through regulation of translation initiation/efficiency and participation in productive complexes (base-pairing and protein/RNA complexes). Some modifications are stable components of long-lived RNAs, e.g., pseudouridines in rRNA molecules and *hTERC*; others are transiently introduced in a specific subset during highly regulated processes (i.e., activation-induced cytidine deaminase function in B cell development [177]). In fact, epitranscriptomic marks are added/removed post-transcriptionally by writer/eraser enzymes and regulate several biological processes, acting as regulatory switches that rapidly change the function of the RNA molecules without requiring new synthesis. RNA modifications may also affect RNA degradation. In recent years, DNA repair enzymes have emerged as factors in RNA metabolism, especially in rRNA biogenesis. It has been proposed that BER enzymes may represent a pathway for targeted recognition of subtle chemical RNA modifications/damages for degradation. One example is represented by SMUG1, which has an RNA-processing function, recognizing hm5U modification in rRNA and in *hTERC* (Figure 7). In the absence of SMUG1, accumulation of hm5U containing molecules was accompanied by increased levels of misprocessed pre-rRNA and a concomitant decrease of the mature rRNA forms [88], suggesting that the recognition of RNA modifications by SMUG1 may be coupled to RNA degradation via the exosome. It is not known how this modification is introduced or generated in RNA, but hm5U occurs on both 18S and 28S rRNAs. One possibility is that hm5U arises from hm5C by spontaneous or enzymatic deamination via apolipoprotein B mRNA editing, catalytic polypeptide-like (APOBEC) enzymes. Future studies are required to pinpoint whether the activity of SMUG1 on RNA processing and maturation has a wider impact on gene regulation.

In summary, SMUG1 and other BER enzymes are emerging as regulators in RNA metabolism and RNA surveillance. The role of SMUG1 in recognizing subtle chemically modified bases could be a key feature of this newly discovered mechanism for distinguishing aberrant RNA from the normal RNA pool. However, the development of new sequencing techniques for hm5U detection and distribution within RNA molecules, is required for better characterization of the combinatorial presence of RNA modifications and their biological significance.

## Data Availability

Not applicable.
